# Changes in Expressions of Spermatogenic Marker Genes and Spermatogenic Cell Population Caused by Stress

**DOI:** 10.3389/fendo.2021.584125

**Published:** 2021-10-11

**Authors:** Pengxiang Tian, Zhiming Zhao, Yanli Fan, Na Cui, Baojun Shi, Guimin Hao

**Affiliations:** ^1^ Department of Reproductive Medicine, The Second Hospital of Hebei Medical University, Shijiazhuang, China; ^2^ Department of Pediatric Surgery, Second Hospital of Hebei Medical University, Shijiazhuang, China

**Keywords:** stress, testis, spermatogenesis, gene, RNA sequencing

## Abstract

Many young adults are in a state of stress due to social and psychological pressures, which may result in male reproductive dysfunction. To provide new insight into this phenomenon, we investigated the effect of stress on the regulation of key genes and biological events in specific stages of spermatogenesis. After establishing rat stress models of different time durations, we observed pathological changes in testis through haematoxylin and eosin staining, and analysed gene expression in testis by RNA-seq, bioinformatic analysis, and reverse transcription qPCR (RT-qPCR). Immunohistochemistry (IHC) with the TissueFAXS quantitative imaging system was used to verify changes of different population of spermatogenic cells marked by differentially expressed marker genes. Our results showed that prolonged stress can lead to pathological changes in the testes, such as thinning of the spermatogenic epithelium, a decreased number of spermatogenic epithelial cells, the disordered arrangement of spermatogenic cells, and a decreased number of mature sperms. RNA-seq revealed that key marker spermatogenesis-related genes such as *Stra8*, *Sycp3*, *Piwil1*, and *Tnp1* had significantly decreased expression levels in chronic stress groups, and this was confirmed by RT-qPCR and IHC. Collectively, these findings suggest that chronic stress causes damaging pathological changes in testis and dysregulates the marker genes of specific stages of spermatogenesis and change the population of spermatogenic cells, which may be a critical responsible for male reproductive dysfunction.

## Introduction

Stress is defined as a real or perceived threat from internal or external adverse stressors to the homeostasis of the body (1). Stress responses can include changes in the neuroendocrine system, autonomic nervous system, and behaviour. These changes can enhance the sensitivity and response efficiency of the body to its environment; however, repeated exposure to stress may produce adverse health effects (2). Of late, as acute and chronic stress conditions have become common due to the fast pace of modern life and an increase in the social pressures and competition that people face, reproductive health problems have become a major problem that troubles people of childbearing age (3). In this context, a biological-psychological-social model of medicine has emerged. The World Health Organization has consequently proposed a new definition of reproductive health: that physical, psychological, and social attributes are in a good condition across all reproductive functions and throughout life.

Reasons for the deterioration of reproductive health are complex (4, 5). The rate of infertility among all couples is 15%, and among these infertile cases, the rate of infertility caused by males is 50% (6). With increasing cases of male infertility in recent years, studying the effects of stress on the male reproductive system has become a focus for medical researchers (3).

Spermatogenesis is a continuous developmental process of germ cells from spermatogonia to spermatids and requires proper function of key genes delicately regulated by some related hormones (3, 7, 8). In the last decade, considerable efforts have been devoted to understanding the molecular feature of the germ cell developmental trajectories (9–11), and some mechanisms such as oxidative stress have been proposed to participate in male infertility (12–14). Although those studies have provided valuable insights, we still know very little about the effect of chronic stress on specific stages of spermatogenesis and on the regulation of key genes and biological events.

Therefore, for the present study, we established rat models of psychosomatic stress and examined the marker genes’ expression levels in spermatogenic cells. Using RNA-seq, RT-qPCR, immunohistochemistry (IHC), and the TissueFAXS quantitative imaging system, our study revealed the hypothesis that chronic stress has an adverse effect on the testicular transcriptome and changes the component of spermatogenic cells, which ultimately leads to male reproductive dysfunction.

## Materials and Methods

### Animals

This study used healthy male Sprague Dawley (SD) rats (Beijing Vital River Laboratory Animal Technology Co. Ltd.), weighing 200 ± 20 g each (8 weeks). The rats (four rats per cage) were placed in an environment with a constant temperature of 22°C, a relative humidity of 50–60% (v/v), and a light/dark cycle of 12/12 h. Experimental rats were fed for 1 week before the experiments. All experiments were approved by the Examination Committee of the Animal Experimental Institution of Hebei Medical University. The experiment was divided into following groups: 3 days, 14 days, and 21 days restraint stress plus ice-water swimming groups (RS + IS) as well as control groups at each time points (eight animals in each group).

### Establishment of the RS + IS Rat Models

RS + IS rat models were established according to published methods (3). On each day, rats in the experimental groups were fixed in the supine position for 6 h (08:00–14:00). The restrained rats were then placed in an ice-water tank for swimming for 5 min. The duration of treatment was 3 days, 14 days, or 21 days. Over these same time periods, rats in the control groups could move freely in their cages but were also fasted and water-deprived. All rats were given food and water *ad libitum* during rest periods.

### Treatment of Rat Testicular Tissues

Sixty minutes after RS + IS, rats were anesthetized and their testes were removed and fixed with 10% formalin. Fixed tissues were then sectioned and stained, following dehydration, clearing, and paraffin embedding. The haematoxylin and eosin (HE) and immunohistochemical staining results were observed by optical microscopy (Olympus IX73; Olympus, Tokyo, Japan). Tissue samples for RNA-seq and RT-qPCR were taken quickly, frozen in liquid nitrogen, and then preserved at −80°C.

### RNA Extraction and cDNA Synthesis

Total RNA was isolated from tissue samples using Trizol reagent (Invitrogen Corp., Carlsbad, CA, USA) according to the manufacturer’s instructions. RNA concentration and purity were determined using a NANODROP 2000C spectrophotometer (Thermo Fisher Scientific, Waltham, MA, USA). For cDNA synthesis, 100–200 ng of total RNA was reverse-transcribed using a quantitative reverse transcription kit (Qiagen, Hilden, Germany).

### Construction of RNA-Seq Libraries

Three testicular samples were taken from each group to construct 12 cDNA libraries. For each sample, 3 μg RNA was used as the starting material. Ribosomal RNA was removed using the Epicentre Ribo-Zero™ Gold kit (Rat) (Epicentre, an Illumina company, Madison, WI, USA). These libraries were constructed based on the recommendations of the NEBNext^®^Ultra™ Directed RNA Library Preparation Kit. RNA fragments and short-stranded RNA were carried using NEBNext First Strand Synthesis Reaction Buffer (5×). First-strand cDNA was synthesized using a random hexamer primer and M-MuLV reverse transcriptase. Subsequently, second-strand cDNA was synthesized using DNA polymerase I and RNase h, the purified second-strand cDNA ends were repaired, and then poly (A) and A adapters were added. A fragment of approximately 300 base pairs (bp) was selected using the UNG enzyme. Clustering was performed in the cBot cluster generation system using the TruSeq PE cluster Kit v4-Cbot-hs (Illumina). After clustering, the libraries were sequenced by the Illumina HiSeq-4000 platform, to obtain 150 bp double-ended sequences.

### RNA-Seq Expression Analysis

The raw reads generated by RNA-seq were processed by removing the Illumina adapters with Trimmomatic, and then low-quality regions at the 5’ and 3’ ends were deleted. The reads (stored in fastq format) were then aligned to the rat (RGSC/rn6) reference genome Rnor_6.0, downloaded from Ensembl (ftp://ftp.bl.org/pub/relee-a-/rattus_norvegicus/), using HISAT2 (15). Sorted bam files were quantified for gene expression using the HTseq-count tool (16), and a counts file was generated. The statistical significance of differences in expression levels between normal and stress samples was calculated using the DEseq2 R package (17) with default parameters. Marker genes of different stages of spermatogenesis were downloaded from GEO (accession: GSE112393 and GSE107664) (18, 19) (http://www.ncbi.nlm.nih.gov/geo/), and screening and clustering analyses were performed. We also performed GO analysis using clusterProfiler (20) tools.

### RT-qPCR

RT-qPCR was performed using the LightCycler 96 real-time PCR system (Roche Molecular Biochemicals, Basel, Switzerland). Each 20 μl reaction contained 1 μl (0.5 μM) of forward primer, 1 μl (0.5 μM) of reverse primer, 6.5 μl of double-distilled H_2_O, 1.5 μl of cDNA, and 10 μl of 2×HI SYBR green qPCR mix (HaiGene). Three biological replicates were tested for each gene, and reactions were run in triplicate. Gene expression levels were quantified using the 2^−ΔΔCT^ method. β-actin was used as an internal reference.

Primer sequences:

rat-Sycp3-For 5′-GTTCATAAAGAGTTTGGAGGAT-3′
rat-Sycp3-Rev 5′-CATTGGAATAGCTGCTACTTAC-3′
rat-Piwil1-For 5′-ATGCCTCTGAAGCTGGCAAT-3′
rat-Piwil1-Rev 5′-TGATGCTGGCAACGAATCCT-3′
rat-Tnp1-For 5′-AGAGGAGGAAGCAAGAGAA-3′
rat-Tnp1-Rev 5′-TTCGTCACAACTGGCATT-3′
rat-Stra8-For 5′-GAGGAGGAAGAGGAGGAAG-3′
rat-Stra8-Rev 5′-GCGGAGATGATGCTGTTC-3′

### Immunohistochemical Staining

After deparaffinisation and microwave antigen extraction, sections were incubated in 3% H_2_O_2_ chilled methanol for 30 min. Tissues were then blocked with 5% (w/v) bovine serum albumin for 1 h at room temperature to avoid non-specific staining, and incubated with rabbit Stra8-specific monoclonal antibody (1:200, Abcam, ab49602), rabbit Sycp3-specific monoclonal antibody (1:300, GeneTex, GTX15093), rabbit Piwil1-specific monoclonal antibody (1:100, ABlonal, A2150), or rabbit-specific monoclonal antibody Tnp1 (1:100, Abcam, ab73135) at 4°C overnight. The sections were then incubated with a biotinylated secondary antibody (ZSGB-Bio), before being treated for 30 min with horseradish peroxidase-binding biotin. Finally, 3, 3’-diaminobenzidine (DAB) was used as a colour-developing agent to visualise antibody localisation. Nuclei were counterstained with haematoxylin.

### Counting of Labelled Cells

Whole testicular sections were scanned using the TissueFAXS quantitative imaging system coupled to a Zeiss^®^ AxioImagerZ2 Microscope (Jena, Germany), and the number of positive cells was counted by HistoQuest^®^ (TissueGnostics, Vienna, Austria). This quantification technique can accurately count the number of positive cells, the positive cell ratios, and the total number of cells in a whole section. This system has been used for tissue cell counting and quantitative analysis in other studies (21, 22).

### Statistical Analyses

Data were imported into SPSS 21.0 statistical software for statistical analysis. Data are expressed as a mean ± SD. The significance testing of RT-qPCR results and positive cell counts in IHC assays were performed by ANOVA. Fisher LSD was used for pairwise comparisons; a result of *P* < 0.05 was considered significant.

## Results

### HE Staining of Rat Testicular Tissues

**Figure 1:**
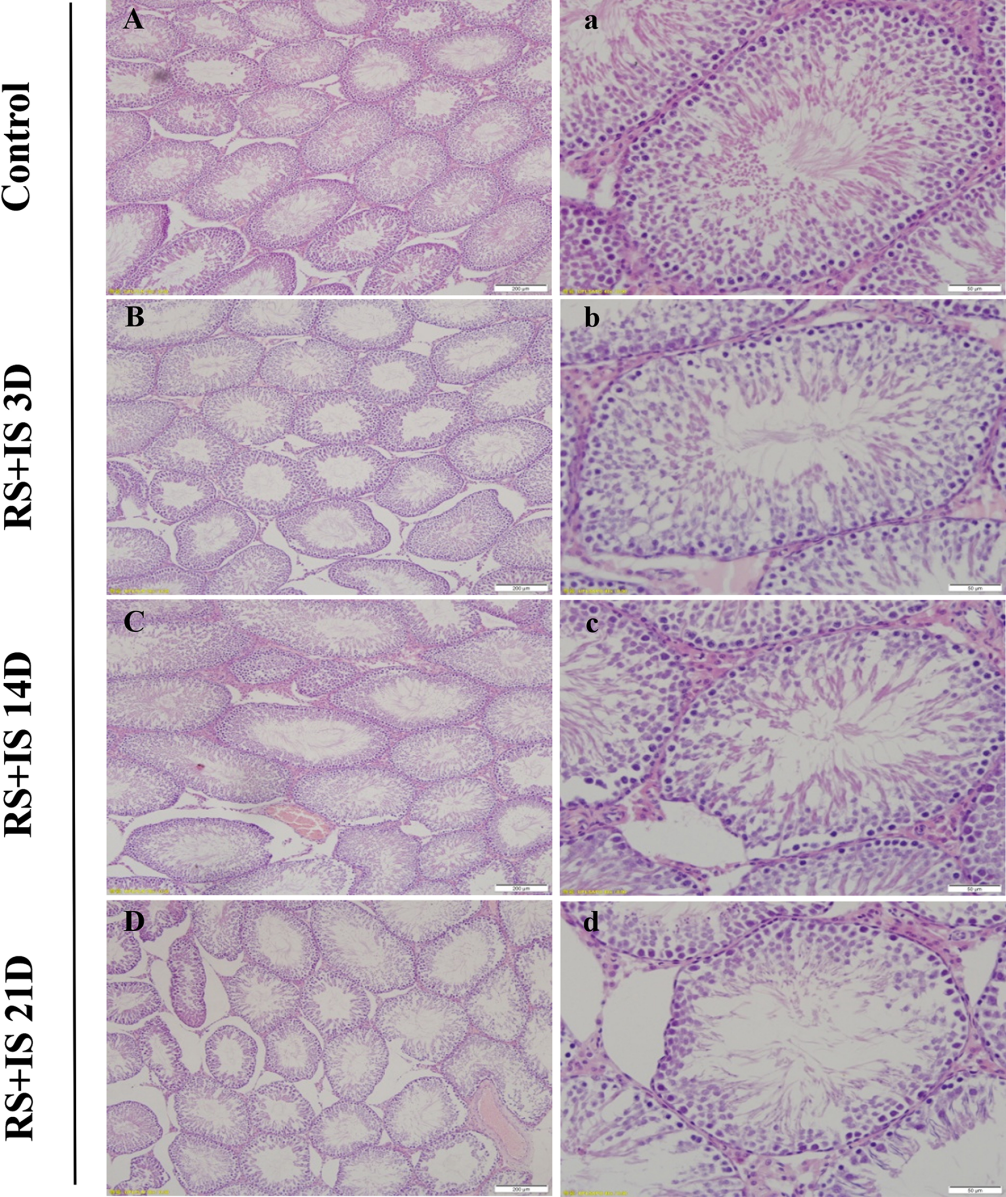
HE staining of the testis. The seminiferous tubules were regular in shape and orderly in arrangement in the control group **(A, a)** and RS+IS group at 3 days **(B, b)**. The tubule basement membrane was separated from spermatogenic cells after 14 days RS+IS exposure **(C, c)**. After 21 days of RS+IS exposure, pathologic changes of the seminiferous tubules were more obvious **(D, d)**; Bars = 200 μm in **(A–D)**; Bars = 100 μm in **(a–d)**.

The testes of rats were insignificant difference between control groups with the passage of time (not shown); testes developed normally and displayed clear structure; the seminiferous tubules and spermatogenic epithelial cells were arranged neatly, and the spermatogenic cells were well-developed (**Figure 1**). In the 3-day stress group, the testicular morphology was similar to that in the control group; however, in the 14-day stress group, the seminiferous tubules were irregular in morphology, the spermatogenic epithelium was thinned, the spermatogenic cell density was sparser, the lumen was enlarged, there were fewer sperm cells, and some of the spermatogenic epithelial cells were separated from the basement membrane. The testicular phenotype of the 21-day stress group was even more aberrant—the spermatogenic epithelium of the testis was thinner, the spermatogenic cell density was sparser, the lumen was further enlarged, the number of sperm cells further decreased, and there was separation of spermatogenic epithelial cells from the basement membrane.

### Expression of Spermatogenic Cell Marker Genes at Different Stages of Spermatogenesis

**Figure 2:**
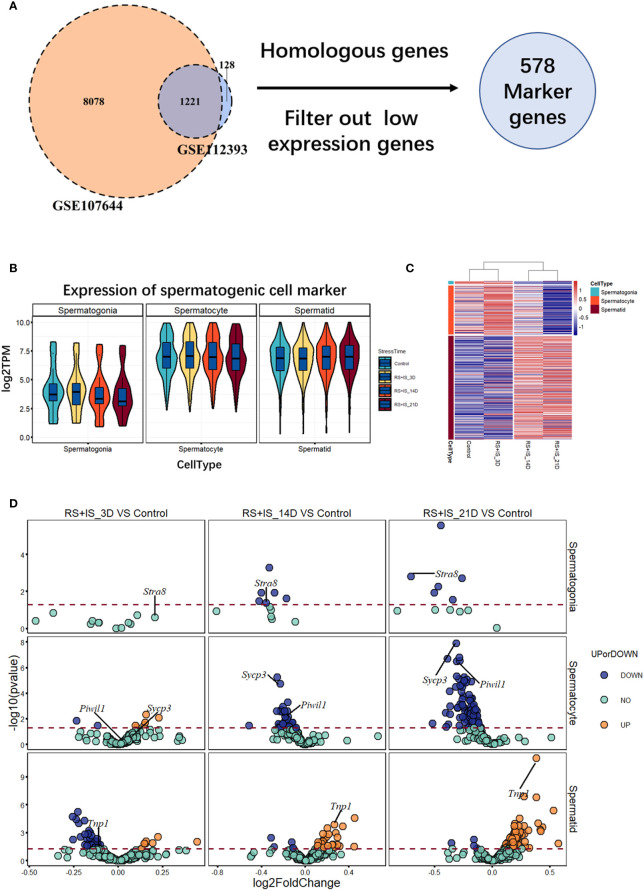
Expression profile of RNA-Seq data between normal and stress group. **(A)** 586 unique homologous markers were found. **(B)** Violin plots show average expression (from three biological replication) value of markers in different durations of RS+IS period. Each Violin plot represents the median, the first quartiles, the third quartiles of gene expression value, and kernel probability density of the data at different values. **(C)** Cluster heat map (from three biological replication) of average germ cell marker expression in different durations of RS+IS period. **(D)** A volcano plot of different expression markers. The horizontal dot line means a p‐value of 0.05. The genes in blue (*P* < 0.05, log2FoldChange < 0) or orange *P* < 0.05, log2FoldChange > 0) are significant different expression markers. No upregulated SPG and SPC markers were found at 14-day and 21-day stress group. Tagged genes were used for further verification.

**Figure 3:**
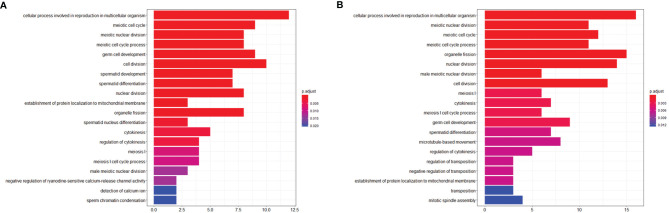
Visualization of Biological process of Gene ontology (GO). **(A)** GO term enrichment of downregulated markers between control group and RS+IS 14-day group. **(B)** GO term enrichment of downregulated markers between control group and RS+IS 21-day group. GO analysis showed significant enrichment terms of downregulated markers, indicating stress affected the normal function of male germ cells.

To investigate the effects of stress on the spermatogenic transcriptome, we used the published single-cell sequencing dataset GSE112393 (18) and GSE107644 (19) to filter our data from different stress durations, and classify them according to sperm developmental stage. These stages were spermatogonia (SPG) marker, spermatocyte (SPC) marker, and spermatid (S) marker (including round and long spermatid). After comparison with single-cell sequencing data and homologous gene dataset (NCBI homologene), 586 unique homologous markers were identified (**Figure 2A**; **Supplementary Table 1**). Violin plots and hierarchical clustering analysis shows the expression condition of all these markers in different duration of RS+IS period (average expression level of samples with log_2_TPM). The result shows SPG marker and SPC marker were significantly downregulated in duration of RS+IS period (**Figures 2B, C**). Clustering analysis also shows the expression pattern of 3-day stress group was similar to control group; 14-day stress group was similar to 21-day group (**Figure 2C**), indicating these spermatogenic cells were in a different physiological and growing inch. DEseq2 was used to analyse differentially expressed genes (DEGs). Ultimately, we found 55 dysregulated genes (14 upregulated and 40 downregulated) in 3-day stress group; 83 dysregulated genes (38 upregulated and 45down regulated) in 14-day stress group; 187 dysregulated genes (86 upregulated and 101 downregulated) in 21-day stress group. In general, we found more downregulated markers than upregulated ones (**Supplementary Table 2**). Furthermore, we found no upregulated SPG and SPC markers in 14-day and 21-day stress group (**Figures 2D**). GO analyses were also performed on significant DEGs (**Figure 3**; **Supplementary Table 3**). All the results indicated chronic stress causes spermatogenic cell dysfunction in transcriptome levels and may lead to spermatogenic dysfunction.

### RT-qPCR of Marker Genes

**Figure 4:**
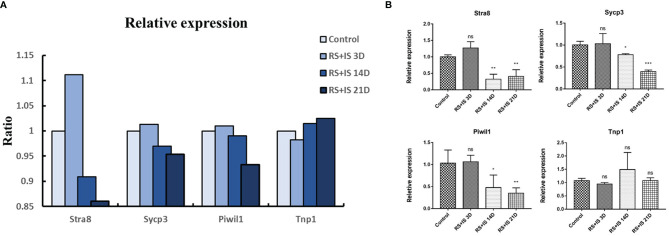
**(A)** Relative expression (TPM) of germ cell marker from RNA-seq. **(B)** Result of RT-qPCR. Data are expressed as the mean ± SD, n = 6. Values with different superscripts indicate significant differences between control and RS+IS groups on one-way ANOVA (^*^ *P* < 0.05, ^**^ *P* < 0.01, ^***^ *P* < 0.001). The mRNA expression levels of Stra8, Sycp3, and Piwil1 were significantly downregulated in chronic stressed groups. ns, non-significant.

To confirm the expression changes identified by RNA-seq, we selected the spermatogonia marker gene Stra8, the spermatocyte marker genes Sycp3 and Piwil1, and the sperm cell marker gene Tnp1 to test by RT-qPCR. The data from the RT-qPCR assays were consistent with the RNA-seq results (**Figure 4**).

### Immunohistochemistry of Marker Genes

**Figure 5:**
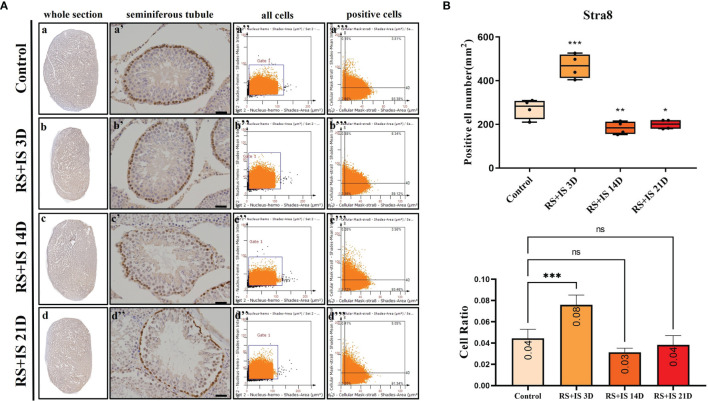
**(A)** IHC expression of Stra8. (a–d) show the section of testis. (a’–d’) show the seminiferous tubule (Bars = 50 μm). (a’’–d’’) positive points represent all cells. (a’’’–d’’’) all the positive points in right upper quadrant represent the positive cells. **(B)** Box plot (upper) shows Stra8-positive cell counting number; each point represents one section. Bar plot (lower) shows Stra8-positive cell ratio (positive cell counting/all cell counting), n = 4. Values with different superscripts indicate significant differences between control and treatment groups on one-way ANOVA (^*^ *P* < 0.05, ^**^ *P <* 0.01, ^***^ *P* < 0.001). ns, non-significant.

**Figure 6:**
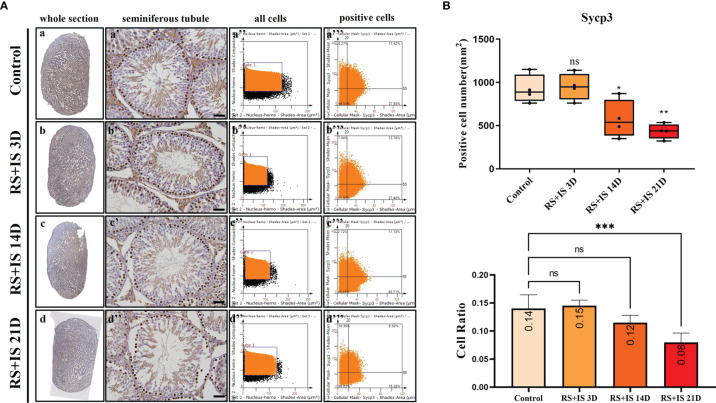
**(A)** IHC expression of Sycp3. (a–d) show the section of testis. (a’–d’) show the seminiferous tubule (Bars = 50 μm). (a’’–d’’) positive points represent all cells. (a’’’–d’’’) all the positive points in the right upper quadrant represent the positive cells. **(B)** Box plot shows Sycp3 (upper)-positive cell counting number; each point represents one section. Bar plot (lower) shows Sycp3-positive cell ratio (positive cell counting/all cell counting), n = 4. Values with different superscripts indicate significant differences between control and treatment groups on one-way ANOVA (^*^ *P* < 0.05, ^**^ *P <* 0.01, ^***^ *P* < 0.001). ns, non-significant.

**Figure 7:**
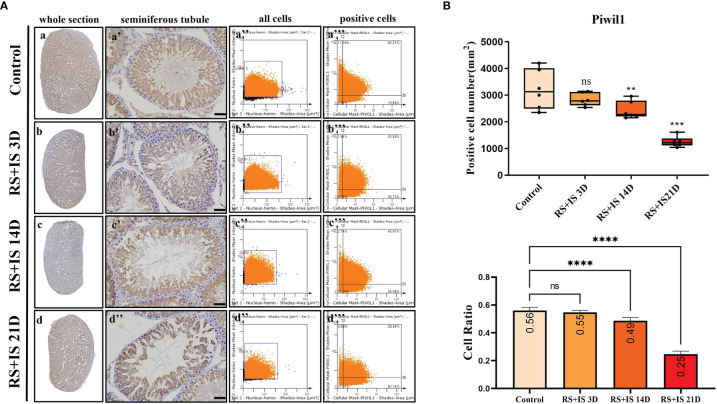
**(A)** IHC expression of Piwil1. (a–d) show the section of testis. (a’–d’) show the seminiferous tubule (Bars = 50 μm). (a’’–d’’) positive points represent all cells. (a’’’–d’’’) all the positive points in the right upper quadrant represent the positive cells. **(B)** Box plot shows Piwil1 (upper)-positive cell counting number; each point represents one section. Bar plot (lower) shows Piwil1-positive cell ratio (positive cell counting/all cell counting), n = 6. Values with different superscripts indicate significant differences between control and treatment groups on one-way ANOVA (^**^ *P <* 0.01, ^***^ *P* < 0.001, ****P < 0.0001). ns, non-significant.

**Figure 8:**
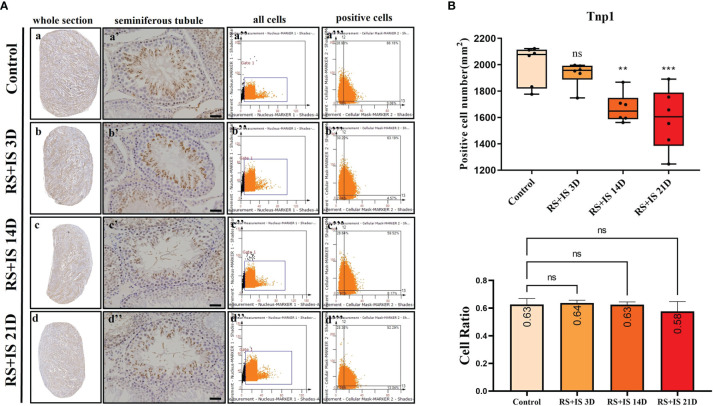
**(A)** IHC expression of Tnp1. (a–d) show the section of testis. (a’–d’) show the seminiferous tubule (Bars = 50 μm). (a’’–d’’) positive points represent all cells. (a’’’–d’’’) all the positive points in right upper quadrant represent the positive cells. **(B)** Box plot shows Tnp1 (upper)-positive cell counting number; each point represents one section. Bar plot (lower) shows Tnp1-positive cell ratio (positive cell counting/all cell counting), n = 6. Values with different superscripts indicate significant differences between control and treatment groups on one-way ANOVA (^*^ *P* < 0.05, ^**^ *P <* 0.01, ^***^ *P* < 0.001). ns, non-significant.

Stra8-positive cells were mainly expressed in spermatogonia near the basement membrane of the seminiferous tubule; Stra8 signal was distributed throughout the nucleus and cytoplasm (**Figures 5Aa’–d’**). Sycp3 was mainly expressed in the nuclei of primary spermatocytes in the seminiferous tubules (**Figures 6Aa’–d’**). Piwil1 was mainly expressed in the primary spermatocytes, in secondary spermatocytes in the seminiferous tubules, and in the cytoplasm of round sperms—with little expression in elongated spermatozoa (**Figures 7Aa’–d’**). Tnp1 was mainly expressed in round sperm and in elongated sperm cells in the seminiferous tubules close to the lumen (**Figures 8Aa’–d’**). Whole testicular tissue sections were scanned and analysed by the TissueFAXS quantitative imaging system; six sections from each group, thus 24 in total. The scatter plots in **Figures 5**–**8** show the total number of cells (a’’–d’’) and the number of positive cells (a’’’–d’’’). Box plot showed that the numbers of Stra8-, Sycp3-, Piwil1-, and Tnp1-positive cells in rat testes in 14-day and 21-day stress groups were significantly reduced compared with the 3-day stress group and control group (**Figures 5**–**8B** upper part). Bar plot showed the Stra8-, Sycp3-, Piwil1-, and Tnp1-positive cell ratio in different durations of stress group; the result shows the changes in the population of cells. The Sycp3-, Piwil1-postive cell ratio was significantly decreased in 14-day and 21-day stress groups (**Figures 5**–**8B** lower part).

## Discussion

Spermatogenesis is a long-range, complex, and delicately regulated process. Developing sperms are sensitive to a variety of external and/or internal stimuli. Previous studies on the effects of chronic stress on male infertility have mainly concerned the reproductive neuroendocrine axes (3, 8), and the techniques are mainly based on animal models, hormone level detection, morphological observation, and animal behaviour (23–26). Although such studies have basically determined that chronic stress has an adverse effect on spermatogenesis, less is known about the effect of chronic stress on specific stages of spermatogenesis and on the regulation of key genes and biological events. The formation of highly differentiated sperm cells from spermatogenic stem cells is a sophisticated process. We hypothesised that the stress response mechanisms of spermatogenic cell types at different developmental stages may vary. In this report, we thus focused on the effect of stress on testicular transcriptomes and described the changes of marker genes of spermatogenic cells in different durations of stress and tracked the cells with differentially expressed marker genes by IHC.

First, based on the successful establishment of rat stress models of different durations, we observed pathological changes in testis tissues using HE staining. Consistent with previous adverse findings (27), we found that with the prolongation of stress time, the number of spermatogenic epithelial cells in rat testes decreased, and pathological changes such as spermatogenic epithelial cell disorder occurred. Second, to further investigate the effect of chronic stress on testis, we conducted studies at the transcriptional level—by using RNA sequencing on testis with different stress duration. We found that the number of genes with changed expression levels increased significantly with the prolongation of stress time, and a GO analysis indicated that these genes are involved in meiosis and germ cell development. These results suggest that chronic stress affects the transcriptome of the testes, mainly manifested in the change of spermatogenic cell population and the dysfunction of spermatogenic cells.

The process of spermatogenesis includes three stages: (1) proliferation and differentiation of spermatogenic stem cells; (2) spermatocyte meiosis; (3) spermiogenesis. Mitosis occurs in the first stage of spermatogenesis, meiosis occurs in the second stage, and spermiogenesis involves a unique morphological transformation. Throughout this complex process, the functions of spermatogonia and spermatocytes rely on specific regulatory genes, which are important to maintain a large number of spermatogenic cells and thereby generate a sufficient amount of sperm (7).

Stra8 is induced by retinoic acid (RA) and is essential for male and female germ cells to enter meiosis. Stra8 mRNA and protein expression is an important and highly specific, highly sensitive marker for the conversion of type A (undifferentiated) spermatogonia to type A1 (differentiated) spermatogonia (28, 29). Sycp3 is the major protein that constitutes the lateral element of synaptonemal complex, which is highly expressed in spermatocytes (30). The correct Sycp3 function is crucial for fertility (31). Piwil1 is specifically expressed in primary spermatocytes, secondary spermatocytes, and round and elongated spermatozoa after the pachytene stage (32). Piwil1 deletion results in the failure of male mouse spermatogenesis (33). Thus, Sycp3 and Piwil 1 are critical for the meiosis of spermatocytes and act as specific markers of spermatocytes. Transition proteins 1 and 2 (Tnp1 and Tnp2) are essential chromosomal proteins that existed before the protamine replacement of histones in the mammalian germline (34, 35). Tnp1 is widely expressed in round and elongated sperm cells and thus can be used as a specific marker for sperm cells. Therefore, for the present study, we used these specific markers to perform verification of the RNA-seq differential expression data through RT-qPCR. The results of these RT-qPCR assays were consistent with the sequencing data.

The testis is one of the most transcriptionally active tissues, exhibiting variable alternative splicing and an uncoupling between transcription and translation (36). To further determine the effects of stress on Stra8-, Sycp3-, Piwil1-, and Tnp1-positive population at different developmental stages, we therefore used the TissueFAXS quantitative imaging system to scan whole testicular tissue sections after immunohistochemical staining. Although Tnp1 RNA had no significant difference after chronic stress on testicular level, Tnp1-positive cell number was significantly decreased. Our result shows the chronic stress decreased the number of Stra8-, Sycp3-, Piwil1-, Tnp1-positive cells. And the ratio of Sycp3-, Piwil1-positive cells declined under chronic stress.

Single-cell RNA sequencing has been greatly developed within the last 5 years. A cornucopia of single-cell transcriptome studies has begun to uncover the full compendium of gene expression map and dynamically revealed some key features of spermatogenesis (37). Due to the high heterogeneity of spermatogenesis and the lack of public single-cell transcriptome dataset of rat testis, our study is limited; however, for exploring differentially expressed genes, bulk RNA sequencing is still an irreplaceable tool (38). Our dataset will provide a reference for future research.

Above all, our findings suggest that chronic stress causes pathological changes of the testis, dysregulates the marker genes of spermatogenic cells, decreases the number of spermatogenic cells, and changes the proportion of spermatogenic cells, which may be critically responsible for male reproductive dysfunction.

## Data Availability Statement

Data availability RNA-seq data are available at the NCBI’s Gene Expression Omnibus (GEO) (https://www.ncbi.nlm.nih.gov/geo/) data repository with the accession ID: GSE144485.

## Ethics Statement

The animal study was reviewed and approved by the Examination Committee of the Animal Experimental Institution of Hebei Medical University.

## Author Contributions

Conceptualization, PT. Data curation, PT, ZZ, YF, and NC. Formal analysis, PT. Methodology, PT and NC. Resources, BS. Software, YF. Supervision, BS and GH. Validation, GH. Visualization, ZZ and YF. Writing—original draft, PT. Writing—review and editing, GH. All authors contributed to the article and approved the submitted version.

## Funding

This study was supported by People’s Livelihood Science and Technology Project of Hebei Province (20377714D), Natural Science Foundation of Hebei Province (H2019206707), and Hebei Clinical Medical Research Center Special Project (20577710D).

## Conflict of Interest

The authors declare that the research was conducted in the absence of any commercial or financial relationships that could be construed as a potential conflict of interest.

## Publisher’s Note

All claims expressed in this article are solely those of the authors and do not necessarily represent those of their affiliated organizations, or those of the publisher, the editors and the reviewers. Any product that may be evaluated in this article, or claim that may be made by its manufacturer, is not guaranteed or endorsed by the publisher.
